# Deviation of the Nail Lamina after Unilateral Partial Matricectomy

**DOI:** 10.3390/healthcare12161681

**Published:** 2024-08-22

**Authors:** Álvaro Saura-Sempere, Rubén Sánchez-Gómez, José Manuel Reguera-Medina, Salvador Márquez-Reina, Rafael Rodríguez-León, Álvaro Gómez-Carrión

**Affiliations:** 1 Podiatry Clinic Álvaro Saura, Mairena del Aljarafe, 41927 Sevilla, Spain; alvarosaura@gmail.com; 2 Nursing Department, Faculty of Nursing, Physiotherapy, and Podiatry, Universidad Complutense de Madrid, 28040 Madrid, Spain; rusanc02@ucm.es; 3 Sanipie Clinic, Utrera, 41704 Sevilla, Spain; josemaremed22@gmail.com; 4 Podiatry Department, Faculty of Nursing, Physiotherapy, and Podiatry, Universidad de Sevilla, 41009 Sevilla, Spain; smarquez1@us.es; 5 GIGI Studios, 08174 Barcelona, Spain; rafrodleo@gmail.com

**Keywords:** nail, foot, surgery, matricectomy

## Abstract

Deviation of the nail plate in the transverse plane has traditionally been regarded as a postoperative complication following the definitive surgical treatment of ingrown toenails, particularly when only a single nail fold is addressed. The quantification and longitudinal comparison of the operated versus non-operated nail folds could elucidate potential transverse deviations of the nail plate. The objective of this study was to assess the presence or absence of transverse nail plate deviation following ingrown toenail surgery on a single nail fold. Methods: A cohort of 11 patients (three males, eight females) with recurrent ingrown toenails undergoing unilateral partial matricectomy were included in this study. Preoperative measurements were compared to those taken at 7, 14, 21, 28, and 35 days postoperatively. Results: The analysis revealed no statistically significant differences in measurements between the operated and non-operated nail folds, nor were there significant changes in the measurements over time within each group (*p* > 0.05). Conclusions: Despite the absence of visible deviations in the orientation of the nail plate, the angular measurements post-surgery at 35 days showed no statistically significant alterations. The angular values observed across all time points appeared to be influenced by the intrinsic morphological characteristics of each nail plate.

## 1. Introduction

Onychocryptosis is a prevalent condition characterized by the interaction of the nail plate with the periungual skin, leading to pain, inflammation, and an increased risk of infection, particularly if not treated in the early stages [1]. This condition most commonly affects the first toe and can present across all age groups [2]. Various classifications have been proposed for its management [3], with surgical intervention being a frequently employed approach [4,5].

Surgical techniques for the resolution of onychocryptosis typically focus on the removal of the offending portion of the nail plate [6]. This is often accompanied by the excision of the corresponding segment of the nail matrix, achieved through mechanical excision [7], wedge resection [8], or chemical ablation techniques [9].

Longitudinal biopsies in the central region of the nail plate can result in chronic dystrophy or even complete detachment of the nail. In contrast, lateral biopsies tend to avoid these complications, though they may lead to onycholysis [10], permanent narrowing of the nail, and potential acquired misalignment [11].

From an anatomical standpoint, 85% of cases involve a ligamentous structure, an extension of the lateral ligament of the distal interphalangeal joint, which originates from the middle phalanx (or distal phalanx in the thumb) and is inserted into the nail matrix and lunula. This ligament might contribute to biomechanical deformation of the nail, possibly explaining certain types of dystrophic nails associated with joint misalignment in fingers and toes [12]. In patients with osteoarthritic changes, alterations at this level could also occur [13].

Physically, there is a correlation between nail shape, growth patterns, and various pathological conditions such as pincer nail and onychocryptosis [14]. However, the aforementioned ligamentous complex is not implicated in any acquired or congenital pathological processes affecting the nail plate [11,15,16,17,18].

Among the secondary complications of surgical treatment for onychocryptosis, acquired lateralization of the nail plate is not widely documented, unlike infection and hemorrhage, which are well-recognized complications [6]. This acquired postsurgical misalignment could potentially lead to poor aesthetic outcomes for patients. Nonetheless, a scarring associated with an excisional matricectomy is currently the primary source of aesthetic dissatisfaction following partial matricectomy, with a chemical matricectomy generally yielding higher satisfaction rates compared to those involving tissue excision [19].

Nail misalignment is a potential complication of extensive excision of the lateral portion of the nail. This misalignment likely arises from a disruption in the forces within the nail unit, affecting both the matrix and the nail plate. The objective of this study is to evaluate the potential for acquired nail plate deviation following surgical techniques for the resolution of onychocryptosis in a single nail fold, particularly when excision of the ligamentous complex is involved.

## 2. Materials and Methods

This study was approved by the Bioethics and Biosafety Committee of the University of Extremadura (CBBUEx) under the approval code ID 13_2023. The ethical guidelines and human research principles outlined in the Declaration of Helsinki were strictly adhered to. All participants were informed of the study’s requirements and provided written informed consent in compliance with Organic Law 15/1999 of 13 December.

### 2.1. Participants

The sample size calculation for this study was performed by the Calculation Center of the Complutense University of Madrid. This study aimed to evaluate the angular differences between the surgically treated nail canals over time. The sample size was determined using G*Power software (version 3.1.9.6, Kiel University, Kiel, Germany). Based on the existing literature indicating that nail plate deviation occurs in 100% of cases following a unilateral matricectomy [5], the calculation was conducted with a 95% confidence interval, a 5% alpha error, and an 80% statistical power with a 20% beta error. A minimum of 8 subjects was calculated to be necessary. To account for potential dropouts, a final sample size of 11 subjects was selected.

The inclusion criteria were as follows: (1) diagnosis of onychocryptosis affecting a single nail edge with a history of previous episodes and no prior diagnosis of onychocryptosis on the opposite edge of the same toe; (2) signed informed consent for the surgical intervention; (3) aged over 18 years. The exclusion criteria included: (1) previous surgical intervention for onychocryptosis; (2) trauma to the treated nail plate at the time of measurement; (3) refusal of the proposed surgical treatment; (4) presence of ungual onychogryphosis; (5) failure to provide signed informed consent for participation in the study.

### 2.2. Instruments, Measurement Procedures, and Variables

For the surgical treatment of onychocryptosis, the excision of the nail matrix was performed using a chemical technique involving phenol, followed by the removal of the phenolized tissue.

Participants were screened for adherence to the inclusion criteria through a face-to-face interview conducted by a member of the research team. The entire procedure was explained in detail, and participants were asked to read and sign the informed consent form before proceeding. Demographic data, including age, height, weight, and BMI, were subsequently collected.

**Figure 1:**
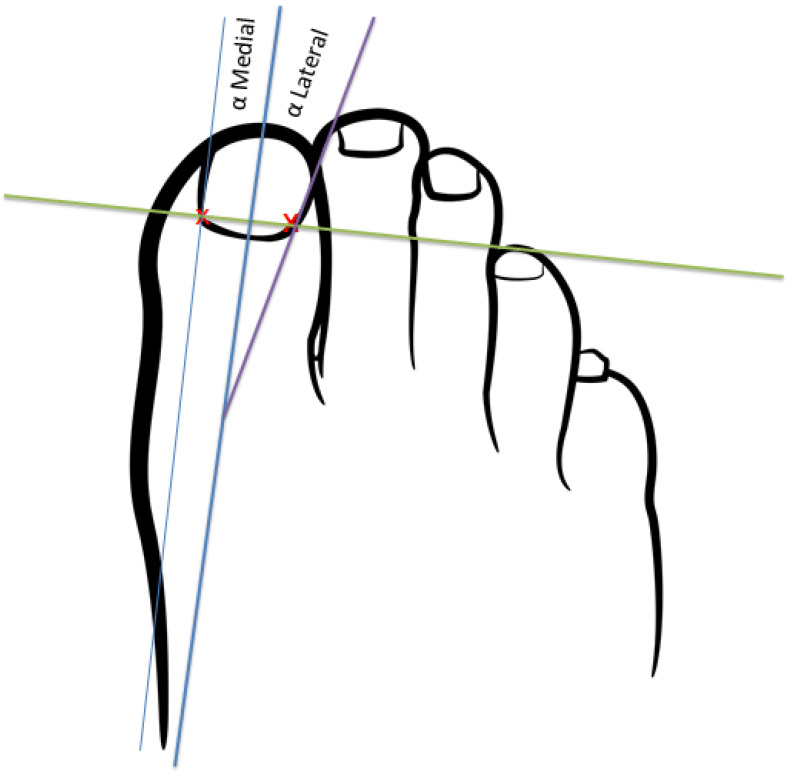
Scheme of the measurements to be taken for the comparison of the intervened and non-intervened nail canals.

**Figure 2:**
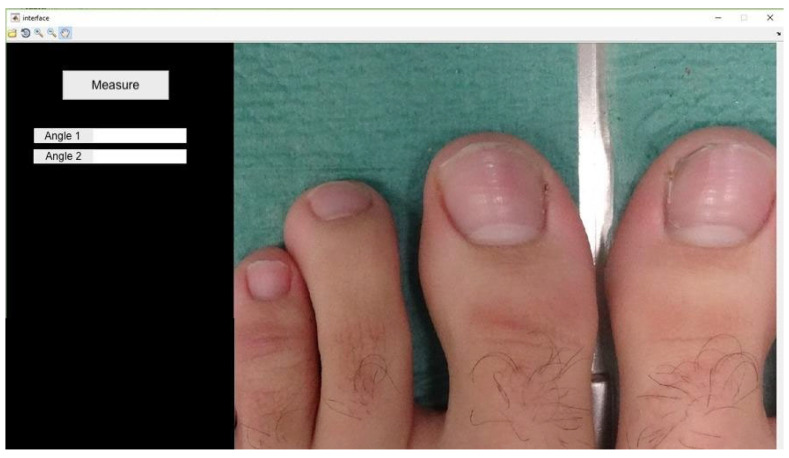
Image taken in constant position according to protocol.

The angle formed by the tangent of the operated and non-operated nail folds relative to the bisector of the nail plate was measured using software developed in MATLAB R2017a, following Pearson*’*s method [20] (Figure 1). Photographs were taken under standardized conditions according to protocol (Figure 2).

**Figure 3:**
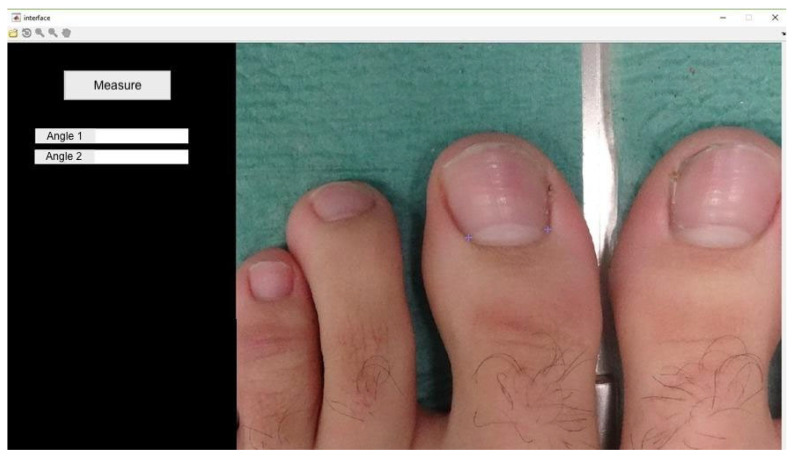
Marking of the first two points (x − x1): termination of the lunula in each nail canal.

**Figure 4:**
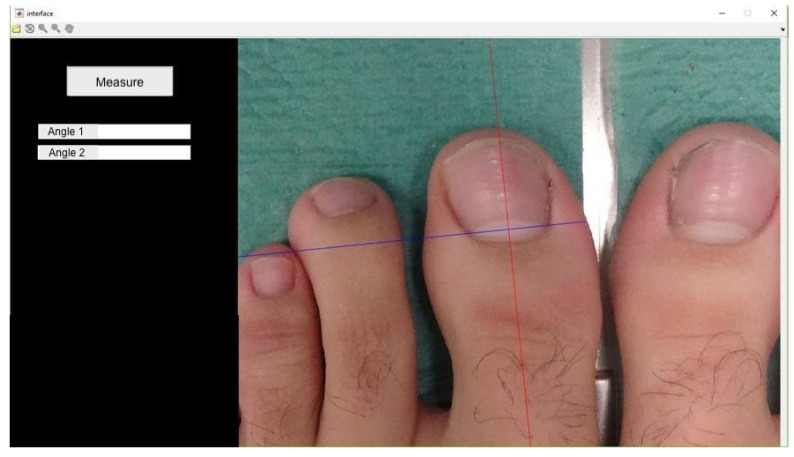
The program automatically determines the line that joins x − x1 and its perpendicular line.

**Figure 5:**
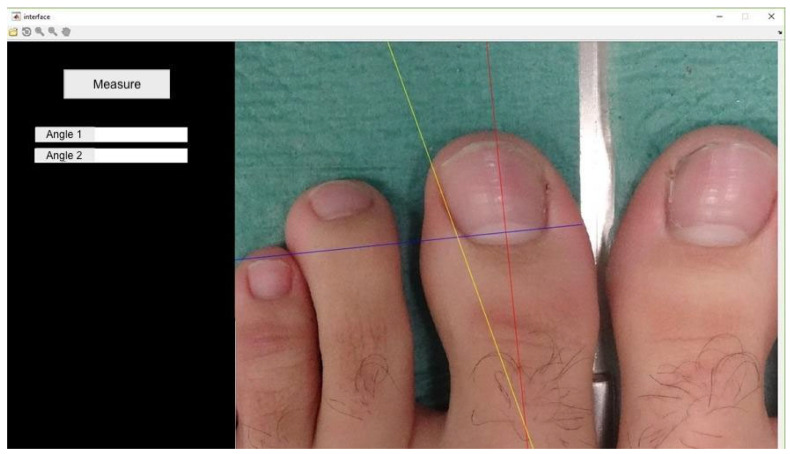
Tracing the tangent line to the lateral nail canal. The angle it forms with the line perpendicular to x − x1 will be angle 1 (lateral angle in this case).

**Figure 6:**
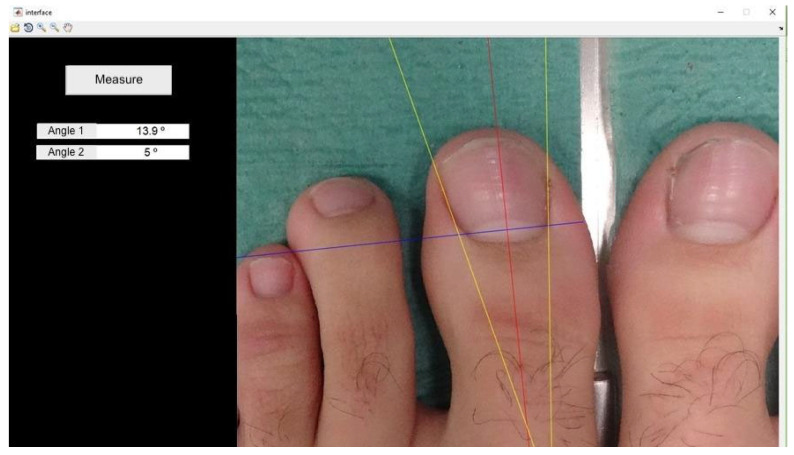
Drawing of the tangent line to the medial nail canal. The angle formed with the line perpendicular to x − x1 will be angle 2 (medial angle in this case).

1. Proximal edge limit point: termination of the lunula in each nail canal (points x and x1) (Figure 3);
2. Bisector of the nail plate (perpendicular line to segment x − x1) (Figure 4);
3. Tangent line to the medial nail edge (Figure 5);
4. Tangent line to the lateral nail Edge (Figure 6);
5. Medial angle: formed by the bisector and medial tangent lines;
6. Lateral angle: formed by the bisector and lateral tangent lines.

Data were collected during the anamnesis process, followed by the capture and measurement of the photographic images. The angle formed by the tangent of the operated and non-operated nail canals relative to the bisector of the nail plate was measured for each hallux.

Measurements were conducted on both pre- and post-surgical photographs, with each angle measured three times to ensure accuracy. This measurement protocol was consistently repeated for 7 days postoperatively.

**Figure 7:**
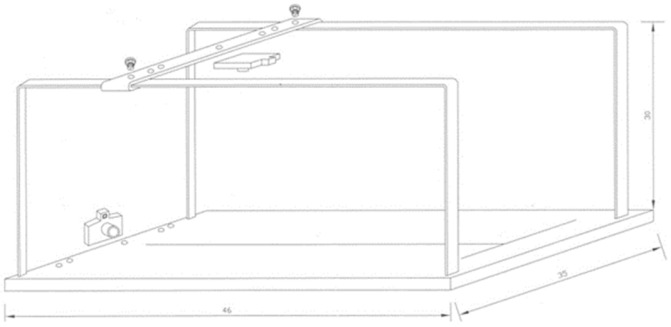
Camera support designed for this study.

Photographs were taken under standardized conditions, maintaining a fixed distance of 30 cm, an inclination of 90°, and a magnification of 3×. A specialized camera support was used to achieve these conditions (Figure 7). The 90° camera inclination was verified for each image captured using a gravitational goniometer.

The data were recorded in an Excel database for Windows 2010 and securely stored on electronic media, in full compliance with current regulations as stipulated in Article 10 of Organic Law 15/1999 on the Protection of Personal Data and Articles 91 and 93 of Royal Decree 1720/2007 concerning security measures for access to personal data in information systems. Post-surgical reviews were conducted according to protocol, with new measurements taken on two occasions: at two and four weeks post-surgery.

### 2.3. Statistical Analysis

To analyze the sociodemographic characteristics of the study population, the mean, standard deviation, and upper and lower limits were calculated for age, weight (kg), height (cm), and Body Mass Index (BMI) (Table 1). The normality of these variables was assessed using the Shapiro–Wilk test (Table 2).

The Intraclass Correlation Coefficient (ICC) was calculated to assess the reliability of the measurements. Values (ICC > 0.9) would indicate the absence of significant errors for each measurement taken (Table 3).

Given that the study involved two groups and aimed to analyze the goniometric characteristics of the measurements between the operated and non-operated groups, independent Student’s *t*-tests were performed. A *p*-value of less than 0.05 (*p* < 0.05) would indicate statistically significant differences between the measurements taken in the operated and non-operated channels (Table 4).

To compare the goniometric characteristics of measurements within the intervened group over time (preoperative and postoperative), paired Student’s *t*-tests were performed (Table 5).

Similarly, the same statistical test was applied to the non-intervened group to compare these measurements in the preoperative and postoperative periods (Table 6). Values (*p* < 0.05) would indicate no significant differences between the measurements taken for each group over time.

## 3. Results

A total of 11 cases of unilateral partial matricectomies were performed, including three males and eight females, for recurrent onychocryptosis affecting a single nail canal. Five cases involved the right foot, while six involved the left. Based on the affected canal, four interventions were performed on the medial canal and seven on the lateral canal (one medial canal on the right foot, three lateral canals on the right foot, four medial canals on the left foot, and three lateral canals on the left foot). The mean age of the patients undergoing surgery was 42.27 years (Table 1).

Measurements taken for each nail canal using the photographic method showed no significant differences between the operated and non-operated nail folds (*p* > 0.05) (Table 4). Regarding angular differences over time between measurements taken for the intervened and non-intervened channels, no statistically significant differences were found (*p* > 0.05) (Table 5 and Table 6). The orientation of the nail plate remained unchanged for 35 days post-surgery. All measurements exhibited angular values characteristic of the morphology of each nail, with no significant differences observed between preoperative and postoperative measurements.

## 4. Discussion

This study aims to evaluate the potential deviation of the nail plate following surgery in a single canal for the treatment of onychocryptosis. Such deviation has been minimally described in the literature as a postoperative complication, though some authors have suggested its occurrence [11,15,17].

Our study results did not reveal statistically significant differences (*p* > 0.05), indicating that the data do not allow us to definitively assert the absence of nail plate deviation following surgical intervention in a single canal for onychocryptosis. Contrary to the observations made by Berker and Baran [11], we did not detect any visually discernible misalignments associated with lateral excisions of the nail plate, even in cases where the excised nail fragment exceeded 3 mm in width. Furthermore, no deviations toward the operated side were observed. The nail plate was sectioned parallel to its bisector, which may have influenced its final visual appearance.

Pearson’s photographic method [20] for assessing potential nail abnormalities suggests that symptomatic onychocryptosis treatment should not be based on correcting a non-existent nail deformity. Therefore, in light of this approach and our study results, we find no evidence to support the systematic association of surgical techniques for onychocryptosis with interventions in both nail canals when only one is affected.

Regarding the potential lateralization of the nail plate as a consequence of resecting the ligamentous complex described by Guéro [12] and supported by Berker and Baran [11], although our study did not produce statistically significant results, we cannot conclusively state that its resection during nail surgery leads to the aforementioned deviation.

As highlighted by Sano et al. [21], cases of nail plate lateralization involve a resultant force imbalance among all structures implicated in the deformity (F ≠ 0), indicating a disruption in the harmony these structures should maintain. This includes the upward mechanical forces, notably the ground reaction force, and the intrinsic forces of the nail unit, such as the extensor tendon of the first toe, nail plate, matriciophalangeal ligament, lateral ligament, and phalangeal-hyponychial ligament.

In surgical cases involving dystrophic or pincer nail deformities [22], the matriciophalangeal, lateral, and phalangeal-hyponychial ligaments, as deformity-maintaining structures, could indeed contribute to lateralization by creating a true imbalance among the involved structures [23]. The valgus component of the first toe could also be an anatomical/biomechanical factor influencing this imbalance. Plantar pressures in patients with nail pathology are slightly lower than in those without pathology at this level [21]. The proprioceptive changes theoretically induced by the absence of a nail plate fragment may also contribute to the mechanical alterations involved in this process.

Future research directions could utilize the method employed in our study to compare nail plate deviations over time with different surgical techniques or to quantify the improvement in certain nail diseases under treatment.

## 5. Limitations

This study has several limitations. The use of an angular measurement program on photographs, even under controlled conditions, presents challenges in achieving precise positioning of measurement points. Additionally, the requirement to take photographs shortly after the surgical procedure may cause inconvenience for the patient. If the surgery proceeds without complications, patients must return for measurements upon surgical discharge.

Moreover, there is a need to extend the data collection period, as we believe that 35 days post-surgery may be insufficient for a comprehensive assessment of potential transverse nail deviation. Another limitation is the small sample size of the study, coupled with the calculation of an 80% power of analysis with a 20% beta error, which must be taken into account.

## 6. Conclusions

Performing unilateral matricectomies for the surgical treatment of onychocryptosis does not exhibit any apparent visual changes suggesting a deviation of the nail plate post-procedure. However, based on the data collected, it is not possible to quantitatively confirm that such deviations do not occur in the transverse plane.

## Figures and Tables

**Table 1:** Sociodemographic characteristics of the study population, according to the division by sex.

Patient	Age	Weight (kg)	Size (cm)	IMC
Media	42.272	70.590	168.000	29.000
SD	19.045	8.683	4.492	4.020
Lim inf	29.48	64.76	164.95	26.30
Lim sup	55.07	76.42	171.02	31.70

Abbreviations: SD, Standard Deviation; kg, kilogram; cm, centimeters.

**Table 2:** Normality Tests of the Variables Studied.

Variables	Shapiro–Wilk *p* Value *
Mean measurement of operated angle day 0	0.364
Half measurement of operated angle day 7	0.463
Half measurement of operated angle day 14	0.320
Half measurement of operated angle day 21	0.307
Half measurement of the operated angle on day 28	0.634
Half measurement of operated angle day 35	0.636
Average measurement of non-operated angle day 0	0.080
Half measurement of non-operated angle day 7	0.106
Half measurement of non-operated angle day 14	0.370
Half measurement of non-operated angle day 21	0.051
Half measurement of non-operated intervened angle day 28	0.638
Half measurement of non-operated angle day 35	0.237

* Shapiro–Wilk Test *p* value.

**Table 3:** Reliability of the variables studied.

	ICC	Lim. Inf–Lim. Sup
Operated angle measurement day 0	0.980	0.943–0.994
Operated angle measurement day 7	0.994	0.982–0.998
Operated angle measurement day 14	0.988	0.966–0.996
Operated angle measurement day 21	0.988	0.967–0.997
Operated angle measurement day 28	0.979	0.941–0.994
Operated angle measurement day 35	0.961	0.893–0.989
Operated angle measurement day 0	0.995	0.985–0.998
Operated angle measurement day 7	0.995	0.985–0.998
Operated angle measurement day 14	0.988	0.966–0.996
Operated angle measurement day 21	0.996	0.990–0.999
Operated angle measurement day 28	0.992	0.979–0.998
Operated angle measurement day 35	0.987	0.963–0.996

Abbreviations: ICC, Intraclass Correlation Coefficient.

**Table 4:** Goniometric characteristics of the measurements of the intervened and non-intervened group.

Goniometric Measurement	Surgical Group (*n* = 11) Media ± SD (IC 95%)	Non-Surgical Group (*n* = 11) Media ± SD(IC 95%)	Valor *p* *
Angle measurement day 0	10.8 ± 6.84 (5.88–15.07)	10.16 ± 7.40 (5.19–15.14)	0.919
Angle measurement day 7	10.08 ± 7.34 (5.14–15.07)	9.07 ± 5.88 (5.12–13.02)	0.727
Angle measurement day 14	10.13 ± 5.93 (6.14–14.12)	10.21 ± 6.59 (5.78–14.64)	0.977
Angle measurement day 21	10.28 ± 5.31 (6.71–13.85)	10.16 ± 7.34 (5.23–15.10)	0.964
Angle measurement day 28	11.25 ± 4.44 (8.26–14.24)	10.83 ± 5.93 (6.85–14.82)	0.854
Angle measurement day 35	10.81 ± 3.93 (8.17–13.46)	10.96 ± 6.20 (6.79–15.12)	0.949

Abbreviations: SD, Standard Deviation; IC 95%, Confident Interval 95%. * Parametric Student’s T for paired samples. Statistical significance for a value *p* ˂ 0.05.

**Table 5:** Goniometric characteristics of the measurements comparing the operated group over time in the preoperative and postoperative periods.

Goniometric Measurement	Media ± SD (IC 95%)	Media ± SD (IC 95%)	Valor *p* *
Comparison of operated angle measurement day 0–day 7	10.48 ± 6.84 (5.88–15.07)	10.16 ± 7.40 (5.19–15.14)	0.477
Comparison of operated angle measurement day 7–day 14	10.16 ± 7.40 (5.19–15.14)	10.13 ± 5.93 (6.15 ± 14.11)	0.977
Comparison of operated angle measurement day 14–day 28	10.13 ± 5.93 (6.15 ± 14.11)	11.25 ± 4.44 (8.27 ± 14.23)	0.877
Comparison of operated angle measurement day 28–day 35	11.25 ± 4.44 (8.27 ± 14.23)	10.81 ± 3.93 (8.17 ± 13.45)	0.319

Abbreviations: SD, Standard Deviation; IC 95%, Confident Interval 95%. * Parametric Student’s T for paired samples. Statistical significance for a value *p* ˂ 0.05.

**Table 6:** Goniometric characteristics of the measurements comparing the group that did not intervene over time in the preoperative and postoperative periods.

Goniometric Measurement	Media ± SD (IC 95%)	Media ± SD (IC 95%)	Valor *p* *
Comparison of non-operated angle measurement day 0–day 7	10.16 ± 7.40 (5.19–15.13)	9.07 ± 5.88 (5.12–13.02)	0.07
Comparison of non-operated angle measurement day 7–day 14	9.07 ± 5.88 (5.12–13.02)	10.21 ± 6.59 (5.78–14.64)	0.109
Comparison of non-operated angle measurement day 14–day 28	10.21 ± 6.59 (5.78–14.64)	10.16 ± 7.34 (5.23–15.09)	0.320
Comparison of non-operated angle measurement day 28–day 35	10.16 ± 7.34 (5.23–15.09)	10.96 ± 6.20 (6.80–15.12)	0.725

Abbreviations: SD, Standard Deviation; IC 95%, Confident Interval 95%. * Parametric Student’s T for paired samples. Statistical significance for a value *p* ˂ 0.05.

## Data Availability

The original contributions presented in the study are included in the article, further inquiries can be directed to the corresponding author.
